# Sleep as a familial and communal matter: a qualitative study of social norms around sleep health in Israel

**DOI:** 10.1186/s12889-023-17003-w

**Published:** 2023-10-24

**Authors:** Dana Zarhin

**Affiliations:** Department of Sociology, University of Haifa, Social Science Building, Mt. Carmel, Haifa, 3190501 Israel

**Keywords:** Sleep health, Social determinants, Social norms, Time and space, Israel, Qualitative research methods

## Abstract

**Background:**

A growing body of research has clarified that sleep is influenced not only by biological factors but also by social factors. While studies have shown that social norms can affect sleep behavior and sleeping arrangements, including when, where, how, and with whom people sleep, researchers still know relatively little about how social norms affect sleep health, especially among adults. The current study explores the association between social norms and sleep health in the Israeli context.

**Methods:**

Data were drawn from semi-structured, in-depth interviews with 66 Israelis—including women and men, Arabs and Jews, and religious and non-religious persons—conducted between February 2020 and February 2022. This article focuses on responses to a set of questions about the comments people make or hear from others about their sleep. Exploring how people comment on the sleep of others highlights prevalent social norms around sleep.

**Results:**

Findings indicate that how sleep is “done” is policed by family and community members who react to norm violations by commenting on what is perceived as “inappropriate” sleep behavior. Comments were made in jest or earnest in response to breaches of social norms regarding sleep timing, duration, continuity, and alertness/sleepiness, indicating that social norms and expectations shape each of these sleep health dimensions.

**Conclusions:**

This article expands the scholarly understanding of the social determinants of sleep health. The study concludes that since individuals may opt to conform to current social norms, which are enforced by members of the family and community, interventions aimed at promoting sleep health should target not only individuals but also the family and community.

## Background

Sleep research has expanded since the 1960s, enhancing our knowledge of many aspects of the subject, including the links between sleep and various individual and public health outcomes [1–3]. Insufficient sleep is associated with increased mortality and morbidity, affecting various dimensions of health, such as cardiovascular, metabolic, immunologic, and mental health [4, 5]. Inadequate sleep often results in tiredness or sleepiness and is associated with occupational and commuting accidents [6–8].

In recent years, sleep scientists have given increased attention to “sleep health” rather than focusing merely on sleep disorders or sleep deficiency [9, 10]. In an attempt to define “sleep health,” Daniel Buysse [9] identified five dimensions that seem most relevant to assessing and measuring sleep:

Sleep duration: The total amount of sleep obtained per 24 h.

Sleep continuity or efficiency: The ease of falling asleep and returning to sleep.

Timing: The placement of sleep within the 24-hour day.

Alertness/sleepiness: The ability to maintain attentive wakefulness.

Satisfaction/quality: The subjective assessment of “good” or “poor” sleep.

These dimensions can be described in positive terms and facilitate a more holistic approach to the study of sleep. They also align with the World Health Organization’s conceptualization of “health” as a “state of complete physical, mental, and social well-being and not merely the absence of disease or infirmity” [11]. Sleep health is a multidimensional concept that directs attention to the socioecological context in which sleep takes place. As Hale et al. [10] note, “sleep health is a function of multiple levels of influence, ranging from individual behaviors to interpersonal factors, community influences, and broader societal influences.” Despite this recognition of the importance of social factors, most sleep research still focuses on biological pathways and mechanisms rather than the sociocultural and physical environments in which research participants live [12].

Still, an emerging body of studies offers important insights. Researchers have identified associations between sleep parameters and sociocultural and environmental factors such as perceived discrimination [13], neighborhood context [14], family context (e.g., parenthood, conflict with a romantic partner) [15, 16], and screen use [17]. In addition, studies have explored associations between sleep beliefs or attitudes and sleep outcomes and behavior [18–20] and have examined the associations between perceived social norms and sleep behavior (e.g., infant sleep practices, use of sedatives and sleeping pills, use of mosquito nets, and sleep-related illness behavior) [21–25]. Nevertheless, researchers still know relatively little about how social norms affect adults’ sleep health. Additional empirical studies are thus needed to elucidate what types of social norms (social rules or expectations) affect the various dimensions of sleep health and how. The current article examines these relationships in the Israeli context.

While the extant body of sleep research has several limitations, a related vein of sociological and anthropological research complements this literature by exploring how sociocultural factors, including social norms, affect sleep. Scholars have explored the different ways in which people around the world have thought about and practiced sleep in various historical periods [26–28], empirically illustrating Simon Williams’s observation that “How we sleep, when we sleep, where we sleep, what we make of sleep, and with whom we sleep, are all socially, culturally and historically variable matters” [29]. For example, the expectation that sleep takes place in the confines of the bedroom (which is currently considered the “appropriate place” for sleep in Western societies) is a result of socio-historical changes: As Norbert Elias’s work illustrates [30], sleep has been civilized in a way that parallels the movement of other “natural” bodily functions from the public to the private sphere. Additionally, scholars [31] have identified three main “sleep cultures”: a monophasic sleep culture in which sleep is consolidated into an eight-hour nocturnal block and daytime sleep is not entirely accepted (prevalent in the United States and Northern Europe); a biphasic sleep culture in which sleep is divided into two primary blocks, a longer one during the night and a short one during the day (a “siesta,” which was once prevalent in Spain and China); and a polyphasic sleep culture in which individuals engage in regulated nighttime sleep but can also sleep at various times during the day. Local beliefs and social norms around sleep also affect decisions about where and with whom to sleep. For instance, whereas Westerners tend to view sleep as a private, individual matter, people in some non-Western cultures [32–36] tend to view solitary sleeping as undesirable because they believe it makes sleepers more vulnerable to various spiritual and/or physical dangers. Thus, people in these cultures prefer to sleep in the presence of others, including family members or even community members.

Furthermore, Simon Williams [29] notes that there exists a “social etiquette of sleep” or a link between normativity and dormativity. Sleep behaviors are open to judgment from others, who might label sleepers as selfish (e.g., when someone sleeps instead of helping their spouse with the housework), attentive (e.g., in certain circumstances where a person is prepared to give up sleep), or stigmatized (e.g., a woman who snores). Meadows et al. [37] contributed to our understanding of the link between normativity and dormativity by empirically examining couples’ dynamics around “bad” sleep behavior. The authors show that breaking civilized codes of conduct in sleep could have biographical or public reputational impacts and cause embarrassment, especially among women. An Israeli study showed that individuals are not entirely exempt from moral responsibility for what happens in their sleep: While they are not blamed for disrupting their partners’ sleep, they are still held accountable and expected to “do something” to diminish their snoring and the disturbance it causes [38]. Additional research on sleep in Israel has highlighted how the local sociocultural context, especially the focus on the family as the core of social life, influences individuals’ sleep practices [39, 40] as well as decisions about whether or not to seek medical care for sleep-related issues [41].

Despite these enlightening studies, there is a need for further empirical research to explore in more depth the sociocultural factors that affect sleep health, particularly how social norms and expectations, and their day-to-day enforcement, shape the various dimensions of sleep health. Such a nuanced understanding would enhance researchers’ knowledge of the social determinants of individuals’ health and well-being, which, in turn, would help researchers and practitioners to design tailored, more effective sleep health interventions.

## Methods

### Sampling and recruitment

This article is based on semi-structured, in-depth interviews conducted between February 2020 and February 2022 as part of a study on the sociocultural patterning of sleep in Israel. Participants were selected using a non-probability purposeful sampling design that sought to include individuals with a wide range of sociodemographic backgrounds in terms of gender, ethnonationality, socioeconomic status, religion, and religiosity, as well as the quality of sleep [42]. The intention was not to create a representative sample but rather to ensure that the sample was homogenous in certain respects (midlife, employed Israeli citizens) to facilitate saturation and heterogeneous with respect to variables deemed analytically important [43].

Individuals had to be between 40 and 60 years old to be eligible for the study. The sample was limited to individuals in midlife because sleep varies across the life course and intertwining sets of (somewhat competing) roles, responsibilities, and expectations are more likely to affect sleep during midlife than at other times [44]. In addition, respondents had to have a job (at least part-time). Sampling working individuals facilitated an examination of the interaction between work and sleep. Respondents with jobs that required regular night shifts were excluded because these jobs differ dramatically from other types of paid work and present unique challenges to individuals and families. To minimize the effect of the spread of COVID-19 in Israel on the results, we suspended the research during lockdowns and resumed it weeks after each lockdown had ended. We also recruited participants who continued to work in their regular jobs and kept their regular schedules despite the pandemic.

Respondents were recruited in multiple ways. Three trained research assistants (RAs) and I created and distributed a flyer describing the research to various Facebook and WhatsApp groups. We also asked friends and colleagues to circulate the flyer to their acquaintances. In addition, the RAs posted the flyer on physical message boards in cities in the northern and central parts of Israel. Finally, we used snowball sampling, although to a limited extent. The flyer included a phone number to allow interested individuals to contact the RAs and inquire about the study. During the resulting phone conversations, the RAs provided additional information about the study and referred callers to an anonymous online questionnaire (written in Hebrew and Arabic) that allowed the research team to assess their suitability for the study. Fifty-four Arabs and 90 Jews completed the screening questionnaire. We approached suitable applicants while ensuring that those we contacted had a range of sociodemographic characteristics to provide a heterogeneous sample, as mentioned above. Recruitment continued until data saturation was reached, i.e., until new interviews did not reveal novel information.

### Interview procedures

Interviews were conducted in places and at times that were convenient to the respondents, such as homes or workplaces, public parks, and coffee shops. Interviews with Jews were conducted in Hebrew and transcribed verbatim, and those with Arabs were conducted in Arabic and translated into Hebrew. I translated the interview excerpts that appear in this article into English. Interviews ranged from 41 min to 2 h and 23 min, with an average of 1 h and 31 min. The semi-structured interviews encompassed a range of open-ended questions about participants’ views and practices of sleep and napping, quality and quantity of sleep, factors that affected their sleep, and more.

This article focuses on a specific set of questions: Participants were asked if people commented on their own sleep and whether they commented on the sleep of others. If they answered yes, they were asked to describe these comments, who made them, and why, and to depict recent conversations on sleep-related matters. The three main prompts and follow-up probes were as follows: Does anyone comment or has previously commented on your sleep? Please tell me about it (Probes: Who made the comment? What did they say? How did you respond? Please describe one of these conversations). Do you comment, or have you commented on someone else’s sleep? Please tell me about it (Probes: What did you say? To whom? Why did you say that? How did they respond? Please describe one of these conversations). Have you talked about sleep with friends, family, or colleagues? Tell me about these conversations.

These questions were chosen to explore how people discuss and respond to others’ sleep and thus shed light on prevalent social norms around sleep. Interviewees were also asked to describe if and how the spread of COVID-19 had affected their experience, management, patterns, or perceptions of sleep in any way. This article focuses on aspects that remained unchanged despite the pandemic.

### Data analysis

Data were collected and analyzed following the principles of constructivist grounded theory, including simultaneous collection and analysis of data, constant comparison, coding, and memo writing [45]. In accordance with the first principle, the research team further explored novel categories and themes that emerged in interviews by adding research questions and probes to the interview guide. Coding of data was done using ATLAS.ti (version 9), a software program designed to assist with qualitative analysis.

The analytic process began with inductive reasoning: I first explored all theoretical possibilities in the data through open coding and then grouped responses that recurred into themes. The initial themes included sleep timing, sleep duration, dozing off, and disrupting sleep. No differences were found between sociocultural groups regarding these themes. The next phase of the analysis focused on abductive reasoning: The iterative process of making sense of the data included moving back and forth between the data and both pre-existing and developing theories [46]. This stage of analysis revealed a strong alignment between the themes that emerged inductively and the dimensions of sleep health identified by Buysse [9]. Throughout the research process, I wrote memos exploring the themes and categories that arose and developed the emerging theory.

### Ethics

This study received ethical approval from the Institutional Review Board at the Faculty of Social Sciences, the University of Haifa (approval number 396/19). Before the interviews, participants were informed of the overarching research goal and the voluntary nature of the interview. All participants signed informed consent forms. Careful measures were taken to ensure respondents’ anonymity and confidentiality, including removing identifying data and using pseudonyms.

### Sample characteristics

The final sample included 66 Israelis aged 40–60 (mean age = 49.48). Twenty-eight respondents were Arab/Palestinian citizens of Israel and 38 were Jews. Since the current study did not ask respondents about their ethnonational self-identification, which prior studies have shown to differ among Arab/Palestinian citizens of Israel [47], the text uses the term “Arabs.” Among the Jews, 18 were religious or ultra-Orthodox, and among the Arabs, 14 were religious. Thirty participants classified their household socioeconomic status as medium-high or high, whereas 36 reported a household socioeconomic status of medium-low or low. The majority were married at the time the research was conducted (n = 54) and had at least one child (n = 62). Concerning sleep quality, 44 participants defined their sleep as good or very good, while 22 defined their sleep quality as bad or very bad. Only 7 participants had sought medical care to treat sleep-related problems, and 6 of those participants had received a diagnosis (obstructive sleep apnea). Finally, 46 respondents described their dwelling as urban, and 19 described it as rural. Table 1 presents the participants’ socio-demographic characteristics.

**Table 1:** Participants’ sociodemographic characteristics

	Value	Arab	Jews	Total
Gender	Male	13	19	32
	Female	15	19	34
Religion	Jews	N/A	38	38
	Christians	7	N/A	7
	Muslims	21	N/A	21
Self-reported religiosity - Arabs	Very religious	0	N/A	0
	Religious	14	N/A	14
	Not religious	9	N/A	9
	Not religious at all	2	N/A	2
	Atheist	3	N/A	3
Self-reported religiosity - Jews	Ultra-Orthodox (*Haredi*)	N/A	9	9
	Religious	N/A	9	9
	Traditional (*Masorti*)	N/A	4	4
	Secular	N/A	16	16
	Atheist	N/A	0	0
Self-reported socioeconomic status	High	3	0	3
	Medium-high	14	13	27
	Medium-low	9	21	30
	Low	2	4	6
Marital status	Married	24	30	54
	Divorced	3	5	8
	Divorced, currently in a relationship	0	2	2
	Widow	1	0	1
	Single	0	1	1
Number of children	0	1	3	4
	1–2	3	11	14
	3–4	23	15	38
	More than 4	1	9	10
Sleep quality	Very Good	3	10	13
	Good	15	16	31
	Bad	0	9	9
	Very Bad	10	3	13
Sought medical care for sleep-related issues	Yes	1	6	7
	No	27	32	59
Dwelling	Urban	17	29	46
	Rural	11	8	19
	Other	0	1	1

## Results

As the inductive analysis shows, how sleep is “done” [48] is policed by significant others who react to norm violations by commenting on what is perceived as “inappropriate” sleep behavior. Most respondents discussed comments they received more than comments they made to others. Respondents heard comments on their sleep from partners, children, and members of their extended families (e.g., parents, siblings, cousins) and friends. Many discussed their comments about their partner’s sleep, but several also mentioned remarks about their children’s sleep. Comments were made in response to breaches of social norms regarding sleep timing, duration, and continuity, as well as alertness/sleepiness, indicating that social norms and expectations shape each of these sleep health dimensions.

### Sleep timing: early/late sleepers and daytime nappers

Although modern Western societies are, to a large extent, non-stop 24/7 societies [49–51], the night is still considered the appropriate time for sleep. However, there appear to be social norms shaping more specific hours as (in)appropriate for sleep.

According to participants’ accounts, one of the most teased sleep behaviors was going to bed at an hour that was deemed early. Early sleepers said they were often the butt of jokes made by family members and friends. Ayesha, an Arab woman, for example, remarked,

Everyone laughs at me for going to bed early. My nephews live in the houses near our house, and sometimes they stay up late, especially in summer, and if they listen to music or something, I call them and say, “You might want to stay up, but others want to sleep.” Sometimes, I shut the windows and turn on the air-conditioner just so that I don’t hear the noise until they go inside, I don’t know precisely when, and then I open the windows again. They always tell me, “You all sleep so early!” [Laughs].

Whereas Ayesha said she and her household members all go to sleep relatively early, other early sleepers alleged they were unique in their own homes. Their partners or children would mock their tendency to go to sleep at hours perceived as too early. Moonia, an Arab woman, recounted:

Everyone constantly jokes that I “switch off” and sleep and that my eyes become red. They have been telling the same jokes about me for 20 years: that I sleep early and cannot stay up till late.

This respondent’s account, along with others, indicated an expectation of sleeping at an “appropriate” time and combatting tiredness or sleepiness successfully. Not adhering to this norm is interpreted as a failure, a comical weakness deserving of mockery. Similarly, Mamdooh, an Arab man, said his friends do not appreciate his early bedtime,

Mamdooh: My friends always comment on my sleep. They say, “You are like the chickens. You are not the only person who has to wake up at 4:30 am. Other people have to wake up at 4:30 am too!”

Interviewer: They say “like chickens” because you go to sleep early?

Mamdooh: Yes, but I don’t care. My body is more important.

This interviewee prioritizes his sleep health but at the social cost of being derided. His account indicates the social expectation to refrain from sleeping at an early hour, even if one has to wake up early in the morning. By mocking early sleepers, significant others conveyed their displeasure with participants’ non-compliance with social norms and consequent premature retirement from social duties and interactions.

On the other hand, several respondents said their family members scolded them for going to bed too late. Comments were made when late bedtime became a habit rather than a one-time thing. Raghad, an Arab woman, recounted,

Sometimes, my family members say, “Enough!” if I stay up working, my husband would say, “Enough! Enough! Go to sleep! Stop working!” He always says, “Stop working, go to sleep. The work never ends.” If we have a messy house and I have lots of housework, then I can’t sleep, but he would say, “Stop that. It will not end. Go to sleep, rest a little, and you can continue doing it tomorrow.”

This respondent’s husband encourages her not to stay up late but does not offer to do the housework. Thus, she continues to go to sleep only after she finishes cleaning the house, even if her husband criticizes her for doing so. Another participant who hangs out with friends several nights a week said his wife reprimands him for going to sleep too late too frequently. Comments about late bedtimes were not made in jest but rather out of concern for sleepers’ health and safety. That is, whereas early sleepers were teased about their inability to stay awake, late sleepers were admonished for surpassing the normative time for sleep. Both early and late sleepers encountered a social response because they breached social norms around what is considered the appropriate sleep timing.

Not only night-time sleep but also daytime napping aroused a social reaction. Despite growing recognition of the benefits of daytime napping [e.g., 52, 53, 54], many still object to this practice. Afternoon nappers described some of the belittling comments they encountered. Abe, a Jewish man, explained:

Abe: I have this thing that I nap in the afternoon for half an hour or an hour. All my friends laugh at me.

Interviewer: Why do they laugh at you?

Abe: They find it strange; but I got used to it. They see 5 pm as the middle of a workday. It’s the end of the workday for me but the middle for them.

As this and other accounts indicate, the workday is often perceived as a time to be awake and productive, and therefore daytime napping is seen by some as a mismanagement of precious time. While several respondents adhered to this view, others said napping after work is a helpful way to manage tiredness and prolong the day to get more things done. Some of these respondents had knowledge of recent medical research that found associations between napping and multiple health and performance gains. However, most nappers emphasized their own experience of the benefits of napping. The variety of views regarding napping was also manifested in respondents’ accounts of how they reacted to their partners’ sleep. Adina, a Jewish woman, shared:

Adina: It really annoys me that my husband sleeps in the middle of the day. He can sleep everywhere. He has no problem with that.

Interviewer: Why does that annoy you?

Adina: Because it’s the middle of the day! Why are you asleep?!

Interviewer: But what is the problem with that?

Adina: It bothers me. Do something else; don’t sleep! [Laughs] I’m talking to him, and he is asleep! He has no problem dozing off everywhere. It’s funny.

This excerpt sheds further light on why some individuals object to daytime napping. Sleep is characterized by reduced consciousness and thus essentially means social absence. A sleeping person is largely (though not entirely [55]) “dead to the world,” relinquishing their social roles and obligations and retiring from social interactions. This absence affects and even offends others who require the sleeper’s conscious presence. Thus, many view sleep as an important and essential (in)activity, but one that has to be limited to certain times at night.

### Sleep duration: short/long sleepers and daytime nappers

Sleep duration is another dimension of sleep health that is shaped by social norms. Both short and long-sleepers recalled getting comments on their quantity of sleep. Nasri, an Arab man, described how he derided his wife for sleeping too much:

I mock her. I have a sense of humor. I tell her, “You are the longest sleeper in the entire world.” I tease her. Sometimes, I drive around the house [during his work hours] at 8 or 9 pm, and I see that everything is turned off. She is asleep! Already in another world! I tell her, “You say you don’t sleep, but you sleep more than everyone else in the entire world. You sleep at 9 pm and wake up at 6 am. You sleep twice as much as my sisters or I do!”

As with this participant, others compared their sleep with that of those around them, especially their partners. Those who believed their spouses slept longer than they did often commented on this. Sometimes they simply said they were envious of their partner’s lengthy sleep, but others mocked their partners for sleeping “too long,” indicating an expectation that sleep should not exceed an appropriate time length. Such excess is seen as a norm violation worthy of social reaction. A few participants explicitly stated that oversleeping is a waste of time, which they do not accept or appreciate.

While long sleepers were laughed at and taunted, short sleepers received grimmer comments, warning of the consequences of their insufficient sleep duration. For example, Dina, a Jewish woman, said,

I comment on my husband’s sleep because I am shocked by how little he sleeps. I am afraid it might hurt his health. He suffers from heart issues, and I don’t know if they’re connected to his short sleep, but I think they are. Along with a proper diet, good, sufficient sleep is vital for maintaining physical balance.

This participant commented on her spouse’s sleep in an attempt to encourage him to extend his sleep duration and thus improve his overall physical health. Like other accounts, this narrative indicates knowledge of the proven associations between sleep and general health. Likewise, Dan, a Jewish man, detailed his wife’s responses to his occasional habit of curtailing his sleep:

Dan: After a night with little sleep, just because I didn’t want to go to sleep, I become tired, exhausted, and then my wife gets upset when I say that I am tired, tired, tired.

Interviewer: What does she say?

Dan: “Enough of that! Why did you stare at your cell phone for an hour?” Or “Why did you surf the internet for an hour and also spent two hours on the computer?!”

This participant’s wife criticized him for using his cell phone and computer at the expense of his sleep, reflecting on sleep hygiene recommendations to avoid screens before bedtime and to prioritize sleep. These examples indicate that some of the medical knowledge about sleep has become part of lay knowledge and perhaps has begun to (re)shape prevalent social norms. In any case, respondents’ accounts indicate that both over-sleeping and under-sleeping are unappreciated and that attempts are made to regulate sleep duration in this sociocultural context.

### Alertness/Sleepiness: dozing off at the “wrong” time and place

Falling asleep outside the bedroom in the presence of others was another experience that aroused teasing. For some, this became a regular occurrence at home or family members’ residences. Although Abeer, an Arab woman, feels sleepy in the early evening hours, she insists on watching some TV with her husband. Yet often she fails to stay awake:

My husband and I watch movies together in the living room, but I fall asleep, so my husband laughs at me, “You barely watched 10 minutes!” Sometimes he would push me gently […] My kids laugh at me too. Sometimes, I go to sleep at around 8 or 9 pm. These are days that I am so tired, but my kids laugh at me when I do that.

In the same way, Yara, an Arab woman, falls asleep not only in her own home but also at her sister’s:

Everyone laughs at me. They know I have a bed ready at my sister’s, and if I stay up, they are all like, “Wow! Yara stayed up today!” Like it’s an achievement. But I can stay up only if I nap in the afternoon. There is no other way that I could stay up after waking at 5 am and running around all day.

As these excerpts clarify, when early sleepers attempt to stay awake, the people around them react in ways that expose social norms and expectations regarding sleep and the body. Praising individuals for staying up later than usual highlights the appreciation of wakefulness and socializing over dormancy. Mocking individuals for failing to stay awake reveals the expectation that a person will control tiredness and drowsiness and overcome sleep. In other words, there is an expectation that the mind will triumph over the body. Inability to control sleepiness and stay alert can sometimes cause embarrassment. As Gabby, a Jewish man, said:

Gabby: Sometimes, I wake up and realize that I dozed off during Friday dinner or when we [he and other family members] sit in the living room together.

Interviewer: Did someone notice you when you dozed off at dinner?

Gabby: Yes.

Interviewer: How did that make you feel?

Gabby: [Laughs] I was a little embarrassed. I made fun of it. They laughed at it.

This interviewee felt embarrassed because he realized he had breached civilized codes of conduct by falling asleep at the “wrong” time and place. His embarrassment indicates the internalization of relevant social norms and expectations.

Respondents’ children were particularly derisive when such norm deviations occurred. Bushra, an Arab woman, and her children watch TV nightly, but despite her best efforts, she keeps falling asleep:

After 9:15 pm, I can’t hold on. They say, “Mom, now you are definitely going to fall asleep” [Laughs]. The other day, my little girl said, “I want to change back to Arabic because you are dozing off, and you don’t explain things to me.” It was about 10 pm.

As with this respondent, others also indicated that their absence due to sleep was commented on as they breached the normative expectation that they stay awake and functional even in the early night hours. Although biologically individuals cannot always resist sleep, norms and expectations demand that individuals avoid such behavior and maintain alertness until specific hours and places considered appropriate for sleep.

Nevertheless, some comments were stern in tone and motivated by concern. Falling asleep outside the bedroom sometimes involved uncomfortable positions, leading people to urge the sleeper to move to the “appropriate” place to sleep. Nadia, an Arab woman, described pleading with her husband to move from the living room to the bedroom when he fell asleep on the sofa at night:

[I tell him], “Get up and move quickly to your bed! Don’t sleep uncomfortably like that. Your body and neck are bent for two or three hours, and then you wake up complaining of neck, shoulder, and arm pain because you sleep inappropriately.”

Similarly, other participants reported making comments that indicated that sleeping outside the bedroom involves “wrong” or “bad” sleep positions that eventually cause pain and may result in weariness. Notably, whereas several respondents had received comments that they looked tired, no respondents described instances in which falling asleep involuntarily was identified as pathological or as requiring medical care.

### Sleep continuity: disruptive sleep behaviors

Respondents reported that they received comments on unruly behavior in sleep (such as hogging of blankets, rolling around in bed, leg movements, etc.) when this behavior disrupted the sleep continuity of those around them. These comments revealed the expectation that sleep should be uninterrupted and consolidated into one nightly block. While this expectation seems reasonable in contemporary Western societies, it would be deemed unreasonable in other sociocultural contexts where punctuated sleep is unavoidable and normalized [36] and in different historical contexts where nighttime sleep was segmented rather than consolidated [56].

The most recurrent sleep behavior that interfered with achieving this goal was snoring, which prevented sleep partners from falling asleep or returning to sleep after waking up. Comments sometimes took the form of mockery, but often they were stern and cautionary. Naif, an Arab man, described how his wife and daughter teased him for snoring:

Naif: They recorded me once.

Interviewer: Who did?

Naif: My oldest daughter and wife. They said, you snored so much, and I responded, “No, I don’t snore. There’s no way” [Laughs].

Interviewer: You denied it.

Naif: Yes, I was kidding around.

Interviewer: So why did they record it?

Naif: My sleep was funny. It was amusing.

While some saw snoring as a simple, even amusing, nuisance, others saw it as a severe problem affecting the health and functioning of the snorer and those around them. Abeer, for example, lamented not being able to share a bed with her spouse (another important social norm [39, 40]). They decided to sleep separately because his snoring disrupted her sleep, causing severe sleep deficiency. She said that after a lengthy period during which she tried to convince him to seek medical aid for his snoring problem, he agreed to see an Ear, Nose, and Throat specialist. As with many other respondents, she recorded her husband to prove her point:

Abeer: At one point, I had to record him so he would hear his snoring.

Interviewer: He denied it?

Abeer: He didn’t realize how bad it was. I had to record him. I still have the tapes. I said, “Do you realize how badly you snore? Do you hear your own sounds? Do you understand that I am worried about you, that you might have sleep apnea?”

Other participants provided similar accounts, indicating that the sound of snoring infringed the social ban against disrupting the sleep continuity of others. Mocking and remarking on snoring were thus social sanctions meant to goad snorers into action, encouraging them to seek solutions for their disruptive sleep behavior, which imperiled themselves and others.

## Discussion and conclusion

This study shows that over and above what prior studies have shown [29, 37, 38], in modern Western societies, dormativity involves multiple facets of normativity, and sleep behavior is constantly regulated. The inductive analysis shows that family and community members react to breaches of social norms and expectations regarding four sleep health dimensions, including the timing, duration, and continuity of sleep, as well as alertness/sleepiness. Thus, this article joins prior studies [57] and enhances the scholarly understanding of the social determinants of sleep.

Specifically, social expectations shape what is considered an early or late bedtime. There are expectations to get what is perceived as sufficient sleep while avoiding over- or under-sleeping, indicating that sleep is seen as a means to an end (good health and function) rather than an objective in and of itself. Too much sleep is considered inappropriate because it is interpreted as social absence. At the same time, too little sleep is unacceptable because it is seen as dangerous both to the short-sleeper and to the people around them. Findings further indicate that there is an appropriate time and place for sleep—certain hours at night and in one’s bed—which makes sleep in other places and times worthy of social reaction. The expectation is that individuals will control their body’s impulses and not give in to sleep. They are expected to stay in command of their bodies, fulfill social roles and duties, and protect social interactions. Yielding to sleep is seen as a display of weakness; though understood, it is frowned upon, causing some embarrassment among sleepers. Even when sleep is voluntary, and despite growing recognition of the benefits of napping [52, 53], many working individuals still judge others by the standards of monophasic sleep cultures (cultures that consolidate sleep into one block at night [27]), rebuking sleep that takes place during the day.

This qualitative study focused on comments that recurred in interviews with this heterogeneous sample of Israelis. While the heterogeneity of the sample increases the likelihood that these findings are generalizable to the general population [58], given the use of a non-probability sampling strategy, the current qualitative study does not aim to generalize the findings to the larger population in Israel. Future quantitative studies should use nationally representative samples to examine to what extent the findings presented in this article are prevalent in the larger population, both in Israel and other countries. Further, additional research should compare how and to what extent different social groups (e.g., Arabs and Jews, individuals with lower and higher socioeconomic status) both conform to and enforce sleep-related social norms in a given context. Additionally, in this case study, respondents commented on four of the proposed sleep health dimensions but not on others (such as regularity or quality/satisfaction); thus, future research should explore whether and how additional sleep health dimensions are regulated in other sociocultural contexts.

This study has practical implications as well. In contrast to multiple health problems that are seen as inevitable, sleep is viewed as a behavior that could be modified, at least to an extent [59]. This is why many sleep associations and specialists lead campaigns and interventions to promote healthy sleep [1, 60–63]. One of the central interventions is sleep hygiene education, which provides a set of behavioral and environmental recommendations designed to encourage healthy sleep habits [64]. These include, for example, going to sleep early enough to get at least 7–8 h of consolidated, uninterrupted sleep, keeping a regular sleep-wake schedule, exercising regularly, and avoiding caffeine [65]. However, these prescriptions for lifestyle changes individualize an issue that, as this study shows, involves more than the individual. Family and community members regulate sleep by calling attention to instances where social norms are breached. Conforming individuals might try to abide by these norms and adopt certain sleep behaviors that could either promote their sleep health (e.g., gaining sufficient sleep) or detract from it (e.g., staying up late despite an early wake time). Therefore, encouraging individuals to implement good sleep hygiene may have limited success if the recommendations provided stand in opposition to social norms. Hence, any intervention aimed at changing individual or public views of sleep or enhancing sleep health should consider these insights and address not only the individual but also the family and community. That is, the findings indicate that educating families and communities on the significance of adequate sleep and engaging them in family-based and community-based interventions will be more effective than targeting individuals. Because sleep contributes to health status [66, 67], improving sleep health will undoubtedly improve public health more generally.

## Data Availability

Research data cannot be shared due to ethical restrictions.

## List of abbreviations

RAs: research assistants

## References

[1] Ramar K, Malhotra RK, Carden KA, Martin JL, Abbasi-Feinberg F, Aurora RN, Kapur VK, Olson EJ, Rosen CL, Rowley JA (2021). Sleep is essential to health: an American Academy of Sleep Medicine position statement. J Clin Sleep Med.

[2] Foster RG (2020). Sleep, circadian rhythms and health. Interface Focus.

[3] Hillman DR, Lack LC (2013). Public health implications of sleep loss: the community burden. Med J Aust.

[4] Grandner MA (2017). Sleep, Health, and Society. Sleep Med Clin.

[5] Grandner MA (2019). Sleep and health.

[6] Vargas-Garrido H, Moyano-Díaz E, Andrades K. Sleep problems are related to commuting accidents rather than to workplace accidents. BMC Public Health. 2021;21(1):1–7. 10.1186/s12889-021-10737-5 PMC8022368 33823824

[7] Lowrie J, Brownlow H. The impact of sleep deprivation and alcohol on driving: a comparative study. BMC Public Health. 2020;20(1):1–9. 10.1186/s12889-020-09095-5 PMC7310070 32571274

[8] Lerman SE, Eskin E, Flower DJ, George EC, Gerson B, Hartenbaum N, Hursh SR, Moore-Ede M (2012). Fatigue risk management in the workplace. J Occup Environ Med.

[9] Buysse DJ (2014). Sleep health: can we define it? Does it matter?. Sleep.

[10] Hale L, Troxel W, Buysse DJ (2020). Sleep Health: An Opportunity for Public Health To Address Health Equity. Annu Rev Public Health.

[11] World Health Organization: Constitution of the World Health Organization—Basic documents. 45th ed. suppl. https://www.who.int/governance/eb/who_constitution_en.pdf

[12] Jackson CL, Walker JR, Brown MK, Das R, Jones NL (2020). A workshop report on the causes and consequences of sleep health disparities. Sleep: J Sleep Sleep Disorders Res.

[13] Grandner M, Hale L, Jackson N, Patel N, Gooneratne N, Troxel W (2012). Perceived racial discrimination as an Independent predictor of Sleep disturbance and daytime fatigue. Behav Sleep Med.

[14] Fuller-Rowell TE, Curtis DS, El-Sheikh M, Chae DH, Boylan JM, Ryff CD (2016). Racial disparities in sleep: the role of neighborhood disadvantage. Sleep Med.

[15] Hagen EW, Mirer AG, Palta M, Peppard PE (2013). The sleep-time cost of parenting: Sleep Duration and Sleepiness among Employed parents in the Wisconsin Sleep Cohort Study. Am J Epidemiol.

[16] El-Sheikh M, Kelly R, Rauer A (2013). Quick to Berate, slow to Sleep: Interpartner Psychological Conflict, Mental Health, and Sleep. Health Psychol (Hillsdale NJ).

[17] AlShareef SM (2022). The impact of bedtime technology use on sleep quality and excessive daytime sleepiness in adults. Sleep Sci.

[18] Baron KG, Gilyard SG, Williams JL, Lindich D, Koralnik L, Lynch EB (2019). Sleep-related attitudes, beliefs, and practices among an urban-dwelling African American community: a qualitative study. Sleep Health.

[19] Nam S, Whittemore R, Jung S, Latkin C, Kershaw T, Redeker N (2018). Physical neighborhood and social environment, beliefs about sleep, sleep hygiene behaviors, and sleep quality among African americans. Sleep Health.

[20] Peach HD, Gaultney JF, Ruggiero AR (2018). Direct and Indirect associations of Sleep Knowledge and attitudes with Objective and Subjective Sleep Duration and Quality via Sleep Hygiene. J Prim Prev.

[21] Hwang SS, Parker MG, Forbes ES, Colvin BN, Colson ER, Brown K (2021). Understanding the barriers and facilitators to safe infant sleep for mothers of preterm infants. J Perinatol.

[22] Kellams A, Hauck FR, Moon RY, Kerr SM, Heeren T, Corwin MJ, et al. Factors Associated with choice of infant sleep location. Pediatrics. 2020;145(3):1–10. 10.1542/peds.2019-1523 PMC7049941 32034081

[23] Lehne G, Zeeb H, Pischke CR, Mikolajczyk R, Bewick BM, McAlaney J, Dempsey RC, Van Hal G, Stock C, Akvardar Y (2018). Personal and perceived peer use and attitudes towards use of non-prescribed prescription sedatives and sleeping pills among university students in seven European countries. Addict Behav.

[24] Perkins J, Krezanoski M, Takada P, Kakuhikire S, Batwala B, Tsai V, Christakis AC, Bangsberg NA (2019). Social norms, misperceptions, and mosquito net use: a population-based, cross-sectional study in rural Uganda. Malar J.

[25] Mulla MM, Lewis JA, Hamilton JC, Tutek J, Emert SE, Witte TH, Lichstein KL (2017). The role of perceived sleep norms in subjective sleep appraisals and sleep-related Illness behavior. J Behav Med.

[26] Rudzik AEF (2015). Sleep around the world: anthropological perspectives. J Roy Anthropol Inst.

[27] Brunt L, Steger B (2008). Worlds of sleep.

[28] Steger B (2017). Japanese historic timescapes: an Anthropological Approach. KronoScope.

[29] Williams SJ (2007). The Social Etiquette of Sleep: some sociological reflections and observations. Sociology.

[30] Elias N (1978). The civilizing process Vol. 1: the history of manners.

[31] Steger B, Brunt L. Introduction: Into the night and the world of sleep. In: *Night-time and Sleep in Asia and the West: Exploring the dark side of life* edn. Edited by Steger B, Brunt L. London and New York: RoutledgeCurzon; 2003.

[32] Lohmann RI. Sleeping among the Asabano: Surprises in intimacy and sociality at the margins of consciousness. In: *Sleep Around the World: Anthropological Perspectives* edn. Edited by Glaskin K, Chenhall R: Palgrave Macmillan; 2013.

[33] Musharbash Y (2013). Night, sight, and feeling safe: an exploration of aspects of Warlpiri and Western sleep. Australian J Anthropol (the).

[34] Brunt L. Sote hue log: In and Out of Sleep in India. In: *Worlds of sleep* edn. Edited by Brunt L, and Brigitte Steger, eds. 2008. Worlds of sleep. Berlin: Frank & Timme. Berlin: Frank & Timme; 2008.

[35] Bird-David N (2017). Us, relatives: scaling and plural life in a forager world.

[36] Hollan D (2013). Sleeping, dreaming, and health in rural Indonesia and the urban U.S.: a cultural and experiential approach. Soc Sci Med.

[37] Meadows R, Arber S, Venn S, Hislop J (2008). Unruly bodies and couples’ sleep. Body & Society.

[38] Zarhin D (2020). You have to do something: snoring, sleep interembodiment and the emergence of agency. Br J Sociol.

[39] Zarhin D (2016). Sleep as a gendered family affair: snoring and the ‘dark side’ of relationships. Qual Health Res.

[40] Zarhin D, Karanevsky-Samnidze A, Aharon M (2022). Co-sleeping with partners and pets as a family practice of intimacy: Israeli couples’ narratives of creating kinship. Sociology.

[41] Zarhin D (2018). Delaying and seeking care for obstructive sleep apnea: the role of gender, family, and morality. Health.

[42] Sandelowski M (1995). Sample size in qualitative research. Res Nurs Health.

[43] Guest G, Bunce A, Johnson L (2006). How many interviews are enough? An experiment with data saturation and variability. Field Methods.

[44] Hislop J, Arber S (2003). Sleepers wake! The gendered nature of sleep disruption among mid-life women. Sociology.

[45] Charmaz K (2014). Constructing grounded theory.

[46] Thornberg R, Charmaz K. Grounded Theory and Theoretical Coding. In: *The SAGE Handbook of Qualitative Data Analysis* Edited by Flick U: SAGE Publications Ltd; 2014: 153–170.

[47] Feinstein Y, Switat MS (2019). Keep a stiff upper lip or wear your heart on your sleeve? Ethnic identity and emotion management among Arab/Palestinians in Israel. Sociology.

[48] Taylor B (1993). Unconsciousness and society: the sociology of Sleep. Int J Politics Cult Soc.

[49] Rogers EL (2016). 24/7: late Capitalism and the ends of Sleep. Dialect Anthropol.

[50] Williams SJ (2007). Vulnerable/dangerous bodies? The trials and tribulations of sleep. Sociol Rev.

[51] Boden S, Williams SJ, Seals C, Lowe P, Steinburg D (2008). The Social Construction of Sleep and work in the British print News Media. Sociology.

[52] Lovato N, Lack L. The effects of napping on cognitive functioning. In: *Progress in Brain Research* vol. 185; 2010: 155–166. 10.1016/B978-0-444-53702-7.00009-9 21075238

[53] Milner CE, Cote KA (2009). Benefits of napping in healthy adults: Rmpact of nap length, time of day, age, and experience with napping. J Sleep Res.

[54] Hayashi M, Motoyoshi N, Hori T (2005). Recuperative power of a short daytime nap with or without stage 2 sleep. Sleep.

[55] Zarhin D. How and why people use Mobile Phones Near Bedtime and in Bed: israelis’ narratives of Sleepful Sociality. Sociology; 2023.

[56] Ekirch RA (2005). At day’s close: night in times past.

[57] Dubar RT (2022). #NoJusticeNoSleep: critical intersections of race-ethnicity, income, education, and social determinants in sleep health disparities. Sleep Health.

[58] Robinson OC (2014). Sampling in interview-based qualitative research: a theoretical and practical guide. Qualitative Res Psychol.

[59] Tubbs AS, Dollish HK, Fernandez F, Grandner MA. The basics of sleep physiology and behavior. Sleep and health. edn.: Elsevier; 2019: 3–10.

[60] American Academy of Sleep Medicine. Sleep well, be well: National campaign makes healthy sleep a priority. https://aasm.org/sleep-well-be-well-national-campaign-makes-healthy-sleep-a-priority/

[61] Albakri U, Drotos E, Meertens R. Sleep health promotion interventions and their effectiveness: an umbrella review. Int J Environ Res Public Health. 2021;18(11):1–39. 10.3390/ijerph18115533 PMC8196727 34064108

[62] National Sleep Foundation. National sleep foundation mission and goals. https://www.thensf.org/mission-and-goals/ Accessed on May 1, 2022.

[63] Zarhin D (2021). Sleep, body work and bodily capital: sleep discourse in the magazines men’s health and women’s Health. Sociol Health Illn.

[64] Hale L, Hale B (2010). Treat the source not the symptoms: why thinking about sleep informs the social determinants of health. Health Educ Res.

[65] American Academy of Sleep Medicine. Healthy sleep habits. https://sleepeducation.org/healthy-sleep/healthy-sleep-habits/

[66] Billings ME, Cohen RT, Baldwin CM, Johnson DA, Palen BN, Parthasarathy S, Patel SR, Russell M, Tapia IE, Williamson AA (2021). Disparities in Sleep Health and potential intervention models: a focused review. Chest.

[67] Grandner MA, Williams NJ, Knutson KL, Roberts D, Jean-Louis G (2016). Sleep disparity, race/ethnicity, and socioeconomic position. Sleep Med.

